# Observation Model for Indoor Positioning

**DOI:** 10.3390/s20144027

**Published:** 2020-07-20

**Authors:** Berthold K. P. Horn

**Affiliations:** Department of Electrical Engineering and Computer Science, MIT, Cambridge, MA 02139, USA; bkph@csail.mit.edu

**Keywords:** Bayesian grid, observation model, transition model, indoor position, indoor location, relative permittivity, fine time measurement, round trip time, FTM, RTT, IEEE 802.11mc, IEEE 802.11–2016

## Abstract

The IEEE 802.11mc WiFi standard provides a protocol for a cellphone to measure its distance from WiFi access points (APs). The position of the cellphone can then be estimated from the reported distances using known positions of the APs. There are several “multilateration” methods that work in relatively open environments. The problem is harder in a typical residence where signals pass through walls and floors. There, Bayesian cell update has shown particular promise. The Bayesian grid update method requires an “observation model” which gives the conditional probability of observing a reported distance given a known actual distance. The parameters of an observation model may be fitted using scattergrams of reported distances versus actual distance. We show here that the problem of fitting an observation model can be reduced from two dimensions to one. We further show that, perhaps surprisingly, a “double exponential” observation model fits real data well. Generating the test data involves knowing not only the positions of the APs but also that of the cellphone. Manual determination of positions can limit the scale of test data collection. We show here that “boot strapping,” using results of a Bayesian grid update method as a proxy for the actual position, can provide an accurate observation model, and a good observation model can nearly double the accuracy of indoor positioning. Finally, indoors, reported distance measurements are biased to be mostly longer than the actual distances. An attempt is made here to detect this bias and compensate for it.

## 1. Background

There has been considerable interest in developing the ability to accurately localize position indoors where GPS can not be used [1,2,3,4,5,6,7,8,9,10,11]. The IEEE 802.11mc WiFi standard provides a protocol for an initiator (cellphone) to estimate its distance from responders (WiFi APs) [12,13,14,15,16,17,18,19,20]. Actually, what is reported is half of the round-trip time (RTT) of an RF signal, multiplied by the speed of light. This *would* be the distance if the signal travelled in vacuum or air in a straight line (and ignoring noise).

Unfortunately, indoors, this simple picture does not reflect reality. First, the estimate of the time of flight is corrupted significantly by a large *position-dependent* error [21] (which may be due to interaction of super-resolution algorithms with fast fading patterns of signal strength) [22].

Secondly, indoors, WiFi signals are slowed down by travel through building materials of high relative permittivity [23,24,25,26,27] (The distance added by an obstacle of thickness *d* and relative permittivity ε is $d(\sqrt{\varepsilon }-1)$). Finally, in some cases, the direct line of sight is blocked by metallic objects or thick layers of absorbing material. In this case the round-trip-time estimate may be based on a signal that has reflected off some surface away from the direct path. In either case, the reported “distance” is larger than the actual distance. Since there is significant bias in the reported distances, one should not expect good results in “multilateration” if one treats reported “distances” as if they were the actual distances.

**Figure 1:**
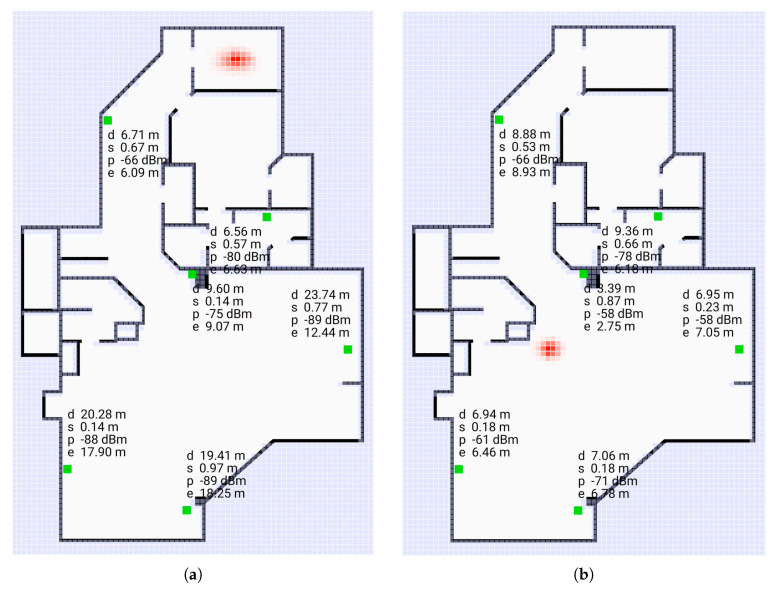
Screen shots of application using Bayesian grid update method as a “heat map” (from [29]). The cells here are 0.25 m on a side. The green spots are the responders. (**a**): initiator not far from centroid of responders, (**b**): initiator outside convex hull of responders. The “hot spot” (red) in (**b**) is larger than the one in (**a**). Note that the paths between initiator and responder typically passes through one or more walls or other obstacles. The grid update method uses the observation model developed here.

Of various methods for estimating the position of the initiator, the Bayesian grid update method [28] has shown promise [21]. Bayesian grid update requires (i) a transition model, and (ii) an observation model. The transition model describes how the initiator may move in the time interval between measurements. The observation model gives the probability of observing a particular *reported* distance conditioned on the *actual* distance between initiator and responder. We show here that a parameterized “double exponential” model fits real data well. Results of using this observation model in Bayesian cell updates are shown in Figure 1 (screen shots from the video [29]).

The accuracy of distance measurement using FTM (Fine Time Measurement) RTT (Round Trip Time) (as per IEEE 802.11mc) is adequate for some applications but not others [21]. Frequency diversity makes it possible to double the accuracy [21], but this is not supported by the current cellphone application programming interface (API). Consequently, increasing the accuracy of position determination by constructing good observation models is important.

We explore the question of the observation model first in a “2-D” setting (single level of a large house), and then in a “3-D” setting (three levels in another house).

## 2. Observation Model

The observation model gives the probability of observing a particular distance conditioned on the actual distance between initiator and responder. If there were no errors, the observation model would be an impulse where reported distance equals actual distance. In the presence of Gaussian measurement noise, the impulse would be spread out into a Gaussian of appropriate standard deviation.

Due to the biases described above, however, an observation model is quite asymmetrical with respect to the peak value. Ignoring effects due to measurement errors for the moment, the reported distance would always be equal to or greater than the actual distance. In the presence of measurement errors, we can expect *some* reported distances to be smaller, but do still expect a rapid drop off from the peak where the reported distance is smaller than the actual distance. Conversely, due to the bias, we can expect a *slow* drop off where the reported distance is larger than the actual distance.

## 3. Building an Observation Model Using Experimental Measurements (2-D Case)

**Figure 2:**
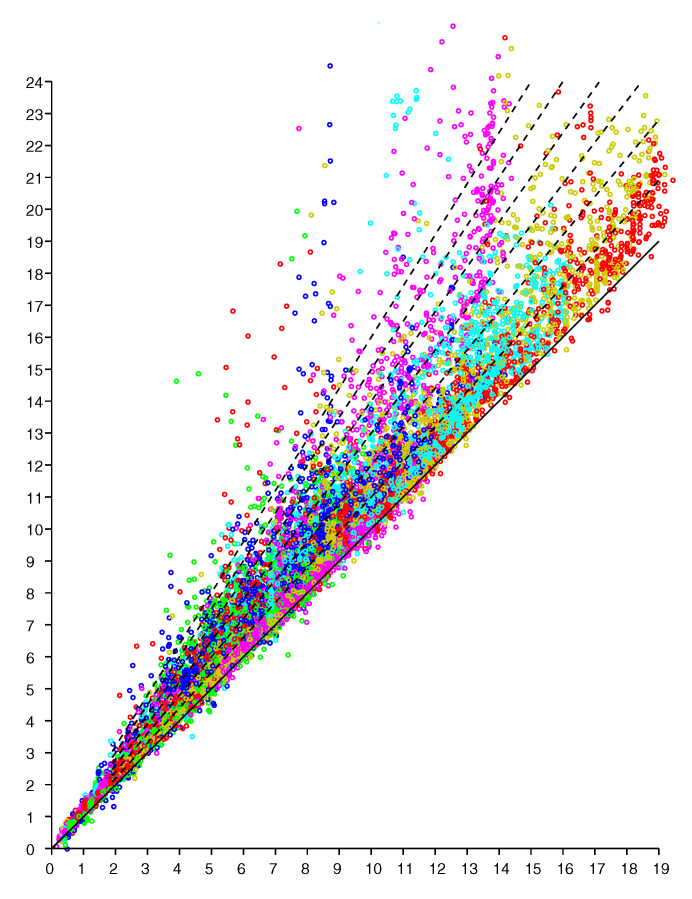
Scattergram of reported distance (vertical axis—meters) versus actual distance (horizontal axis—meters). Results from six responders color coded. (Dashed lines have slope 1.0, 1.1, 1.2 *…* 1.6 ).

An observation model can be based on scattergrams of reported (observed) distance for known actual distances. We can obtain NM data points if we have *N* responders in known positions and take measurements of distances from *M* known positions for the initiator (see e.g., Figures 8 and 9 in [21]). Here we start with a planar “2-D” example of a single level of a large house. The scattergram in Figure 2 shows reported distance versus actual distance. (The average measurement offset of this initiator/responder system has been determined and subtracted out [21]). Such scattergrams can be used to estimate parameters of the underlying probability distribution.

Conveniently, experimental results indicate that the scattergram has structure that allows reduction of what appears to be a two-dimensional probability density distribution fitting problem to a one-dimensional one. In particular, note that in the above scattergram, the vertical spread in reported distances increases more or less in proportion to the actual distance.

**Figure 3:**
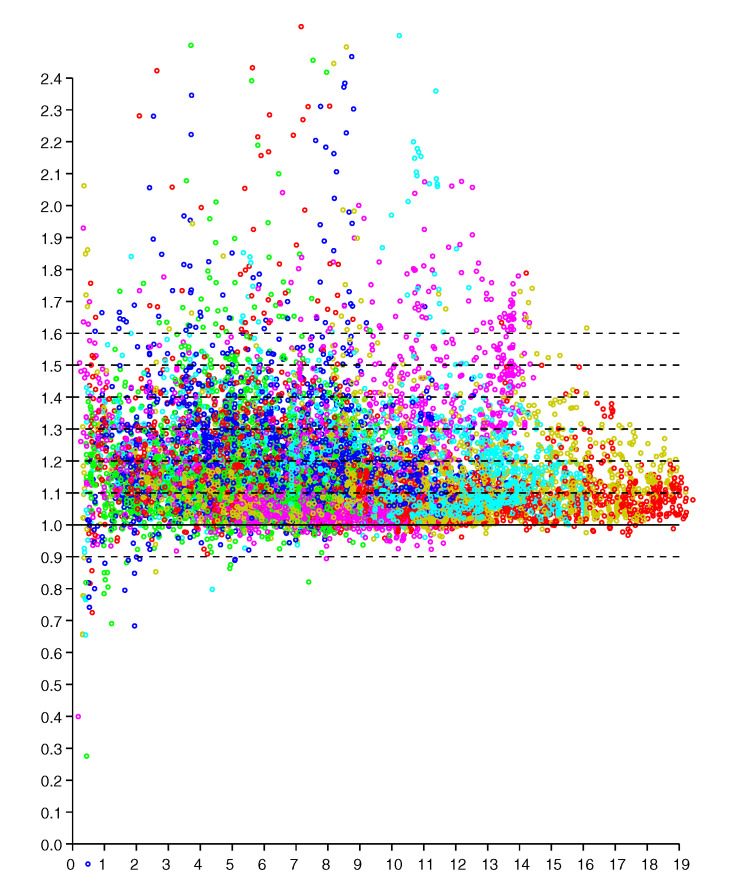
Scattergram of *ratio* of reported to actual distance (vertical axis) versus actual distance (horizontal axis—meters). Results from six responders color coded. (Dashed lines are shown for ratios of 0.9, 1.0 1.1, 1.2 *…* 1.6).

This regularity becomes more obvious if we plot the *ratio* of reported distance to actual distance (versus actual distance), as in Figure 3. The vertical scatter in this diagram is more or less independent of actual distance (except perhaps for very small and very large distances). So we can collapse the two-dimensional scattergram into one were we plot the *ratio* of distances versus the actual distance. It is, of course, easier to fit one-dimensional distributions than two-dimensional distributions, particularly when the number of available samples is limited. This approach has been used before to obtain a piece-wise linear approximation of an observation model from relatively few measurements (see Figure 10 in [21]).

**Figure 4:**
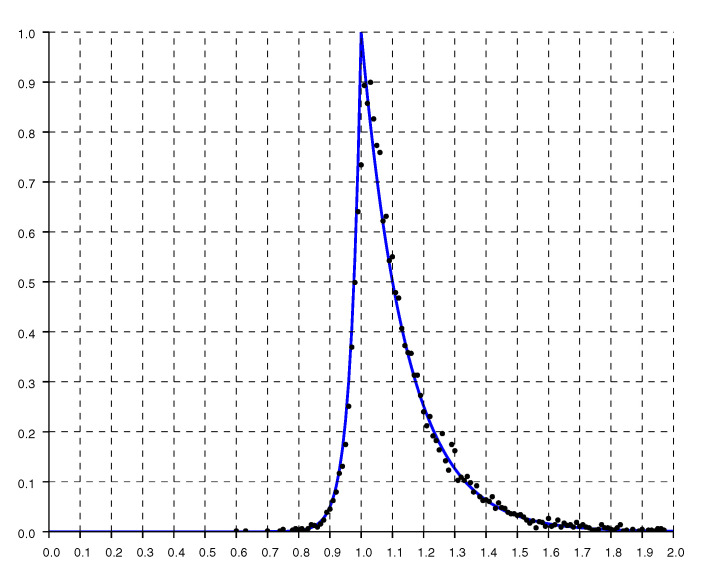
Histogram of the ratio of reported to actual distance based on about 10,000 measurements (scaled so that the peak equals one). The solid line is the “double exponential” parametric fit.

The shape of the histogram becomes clearer when we have many measurements. Figure 4 shows a histogram of the ratio of reported to actual distance as a function of actual distance for about 10,000 measurements (collected in about 10 min). These measurements are from a single level of a house of mostly wooden stud and dry wall construction, but with some heavily tiled bathroom walls, some concrete support beams, and metallic obstacles such as a large refrigerator, an oven and stacked microwave. Here, the asymmetry is quite apparent, with ratios greater than one occurring much more frequently than ratios smaller than one (From the figure that follows we see that about 85% of the ratios are larger than one in this case).

**Figure 5:**
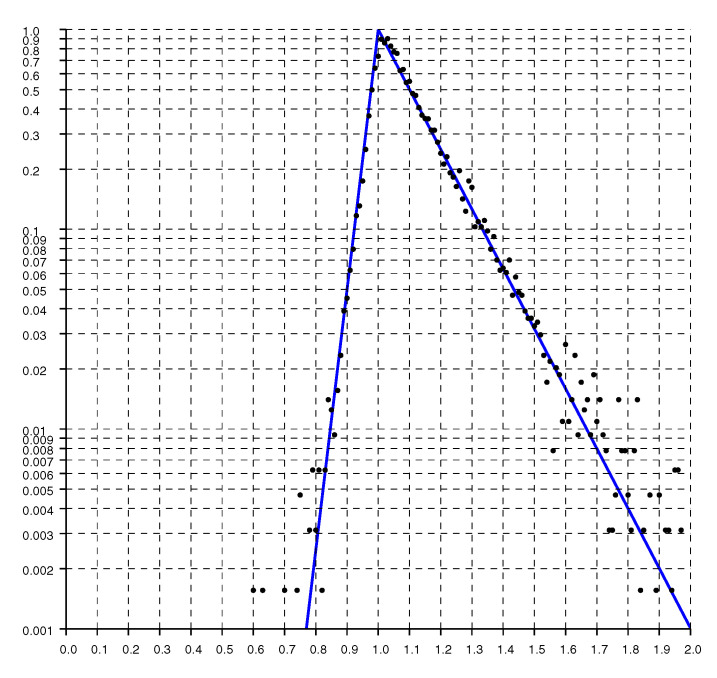
Histogram of the ratio of reported distances to actual distance on a logarithmic scale. The solid line is the “double exponential” parametric fit.

Replotting the histogram on a logarithmic scale, as in Figure 5, shows that the drop offs on either side of the peak are exponential. As expected, the decay is much more gradual for ratios larger than one than it is for ratios smaller than one.

**Figure 6:**
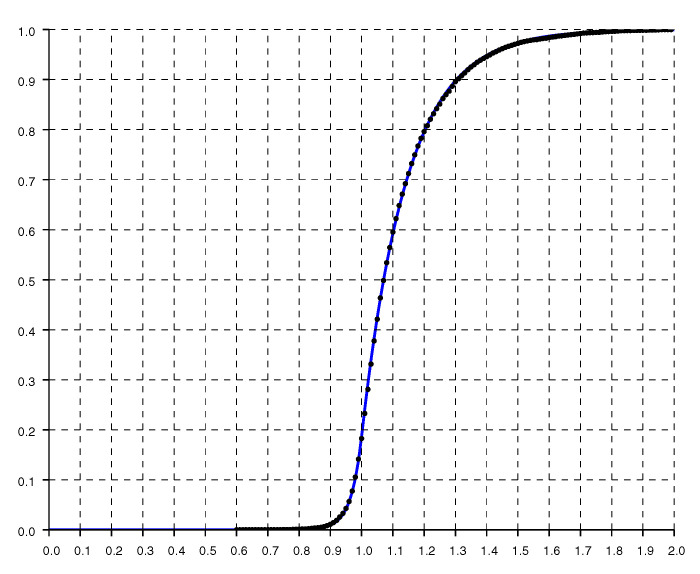
Normalized cumulative histogram of the ratio of reported to actual distance. The solid line is the “double exponential” parametric fit.

A model fit to the histograms from these experimental results is the “double exponential”

(1) $$h\left(r\right)=\frac{1}{H}\left\{\begin{array}{cc} e^{-\left(1-r\right)/s_{l}} & \mathrm{for}\;r<1\\ e^{-\left(r-1\right)/s_{r}} & \mathrm{for}\;r>1 \end{array}\right.$$

where r=o/d is the ratio of, *o*, the reported (observed) distance, to, *d*, the actual distance (with $s_{r}>s_{l}$). The constant $H=s_{l}+s_{r}$ normalizes the distribution so its integral (w.r.t. *r*) equals one. For the solid curves plotted in Figure 4, Figure 5 and Figure 6, $s_{l}=0.033$ and $s_{r}=0.145$.

In terms of h(…), the conditional probability of reported distance, *o*, when the actual distance is *d*, is

(2) $$P\left(o|d\right)=\frac{1}{d}h\left(\frac{o}{d}\right)$$

Another check on the quality of the parametric fit is given by the cumulative distribution shown in Figure 6, where the dots are from experimental data while the solid curve is from the double exponential fit.

The parameters, $s_{l}$, $s_{r}$, estimated from the scattergram, depend on the structure of the building and the building materials. Fortunately, experiment indicate that they need not be known with great accuracy to get reasonable results with the Bayesian grid update method. The key is to get about the right amount of asymmetry.

An aside: In practice a weighted sum of this distribution and a uniform distribution should be used to account for the appearance of random outliers in the measurements.

The model could be further refined by breaking down the scattergram into sections based on distance, since the best-fit parameters seem to depend somewhat on distance. The best-fit $s_{l}$ appears to go down with distance, while $s_{r}$ may go up a bit. This refinement does not change the behavior of the Bayes cell update significantly, and is not needed to get good results.

## 4. Using Calculated Position of Initiator as a Proxy

We need to know the actual distances between initiator and responders when fitting a parameterized model to measurement data. Now, in order to use the method described here for indoor positioning, the positions of the responders already have to be known. We can calculate the actual distances between the initiator and each of the responders, as required for the model fitting, if the positions of the initiator while taking data are also known. While one can certainly set up an experimental situation where the position of the initiator is known accurately, this approach tends not to scale well (unless some independent method for automatically determining indoor position can be found). A manual measurement process as used in [21] is not very practical if we want tens of thousands of measurements.

It may make sense then to use a proxy for the true initiator position if a good enough one can be found. For this purpose, Bayesian grid update can be used to estimate the position of the initiator. The quality of this proxy measurement increases with the number of responders, so this is best done with more than the minimum number of responders needed to solve the multilateration problem In the 2-D case, for example, three responders are the minimum needed for unambiguous solution [21], but it is better to use more. Six responders are shown in Figure 1, which was the setup for data collection, when three responders would have been enough to cover most of the area for 2-D indoor positioning purposes. (Similarly, nine responders are shown, when four responders would have been enough for 3-D indoor positioning).

The resulting scattergram can be used to fine-tune the observation model. The new observation model can then be used in turn for another round to further refine the parameters of the observation model (no need to take new data).

**Figure 7:**
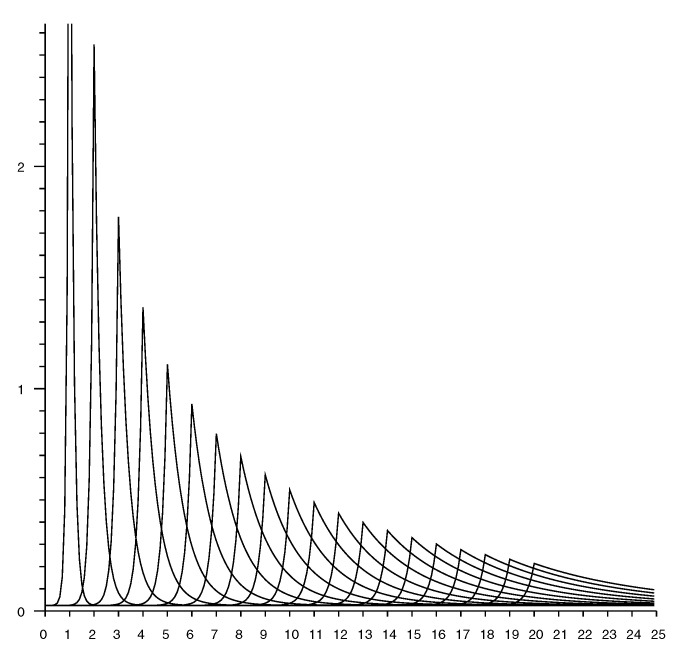
Conditional probability of observing a distance measurement (horizontal axis—meters). Each curve corresponds to a different value for the *actual* distance. Curves shown for actual distance of 1 m, 2 m, *…*, 20 m.

Figure 7 shows slices through an observation model. Each curve shows the conditional probability of a particular measured distance given the actual distance. The plots are for actual distances from 1 to 20 m in 1 m increments.

### Accounting for Random Measurement Error

**Figure 8:**
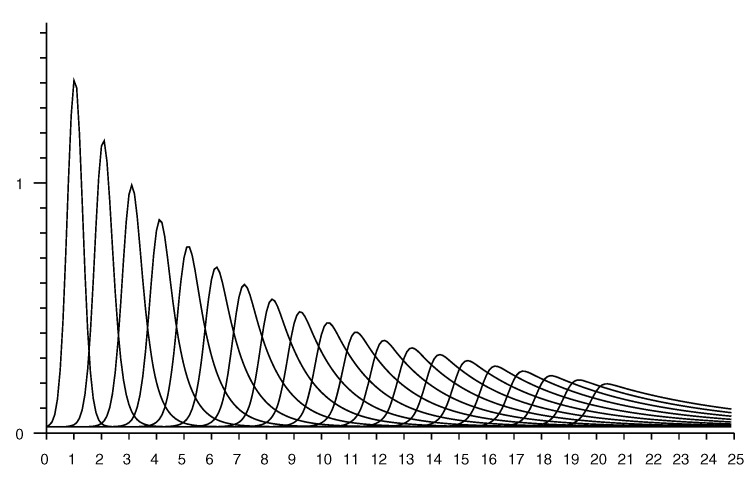
Conditional probability of observing a distance measurement (horizontal axis—meters) taking into account random measurement error with st. dev. of 0.25 m. Each curve corresponds to a different value for the *actual* distance. Curves shown for actual distance of 1 m, 2 m, *…*, 20 m.

The estimate of the conditional probability (from Equation (2)) has peaks that get sharper the smaller the actual distance. In practice, additive random measurement error will smear out such peaks. This can be modelled by convolving the distribution with some local averaging kernel, such as a Gaussian (see Appendix D). The standard deviation of the kernel can be chosen equal to the reported standard deviation from repeated measurements taken in the same position. This “smearing” has little effect on conditional probabilities for longer distances, but will smooth out unrealistically sharp peaks that would otherwise occur for small distances. See e.g., Figure 8.

## 5. Efficient Use of the Observation Model in Bayesian Grid Update

The Bayesian grid update requires stepping through all of the cells of the grid and determining the conditional probability that one would observe the distance reported given the known actual distance of each cell from the responder.

In detail, the whole grid is updated when a reported distance measurement *o* is received relating to responder *i* at $x_{i}$. For each cell in the grid, corresponding to its position x, one first computes the distance from the responder $d=∥x-x_{i}∥$. The probability currently stored in the cell corresponding to x is then multiplied by the conditional probability P(o|d) (After sweeping through the grid in this fashion, the values can be renormalized so that they add up to one).

An aside: One must be careful not to let the limitations of floating-point multiplication produce an actual zero, since once a value is zero it cannot become non-zero again in the multiplicative Bayesian update process.

After the update, if a specific position is needed for the initiator, rather than a probability distribution, then one can use either the peak (maximum likelihood) or the centroid (expected value) of the distribution.

To speed up the above sweep, a “rate vector” can be precomputed when an observation becomes available and used for the whole grid. The rate vector gives the probability that the reported distance, *o* could correspond to the actual distance, *d*, that is,

(3) $$r_{o}\left(d\right)=P\left(o|d\right).$$

Precomputing this rate vector can speed up the Bayesian cell update if the computation of the conditional probability P(o|d) is non-trivial. This is particularly helpful in the case of a 3-D grid of voxels where the number of elements in the rate vector can be much smaller than the number of cells in the grid.

**Figure 9:**
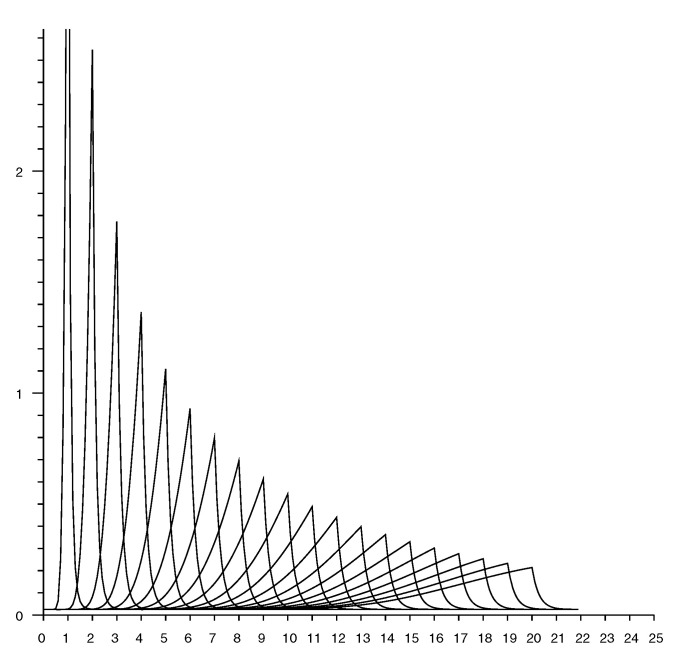
Rate vectors used in updating the Bayesian grid (horizontal axis actual distance—meters). Each curve corresponds to a different value for the *reported* distance. Curves shown for reported distance of 1 m, 2 m, *…*, 20 m.

Figure 9 shows a set of “rate vectors” plotted versus actual distance for reported distances in the range 1 to 20 m in 1 m increments. (Note that the horizontal axis in Figure 7 shows reported distances, while the horizontal axis in Figure 9 shows actual distances).

**Figure 10:**
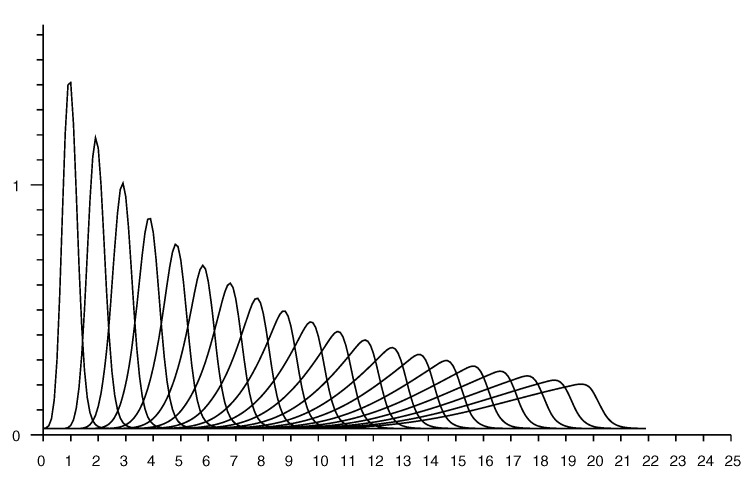
Rate vectors used in updating the Bayesian grid (horizontal axis actual distance—meters) taking into account measurement error with st. dev. of 0.25 m. Each curve corresponds to a different value for the *reported* distance. Curves shown for reported distance of 1 m, 2 m, *…*, 20 m.

As mentioned above, one should take into account the “smearing” effect of measurement errors on the conditional probabilities. Figure 10 shows the rate vector based on the smoothed conditional probability shown in Figure 8 (Note that this is *not* the same as convolving the rate vector itself with the smoothing kernel).

## 6. Building an Observation Model Using Experimental Measurements (3-D Case)

**Figure 11:**
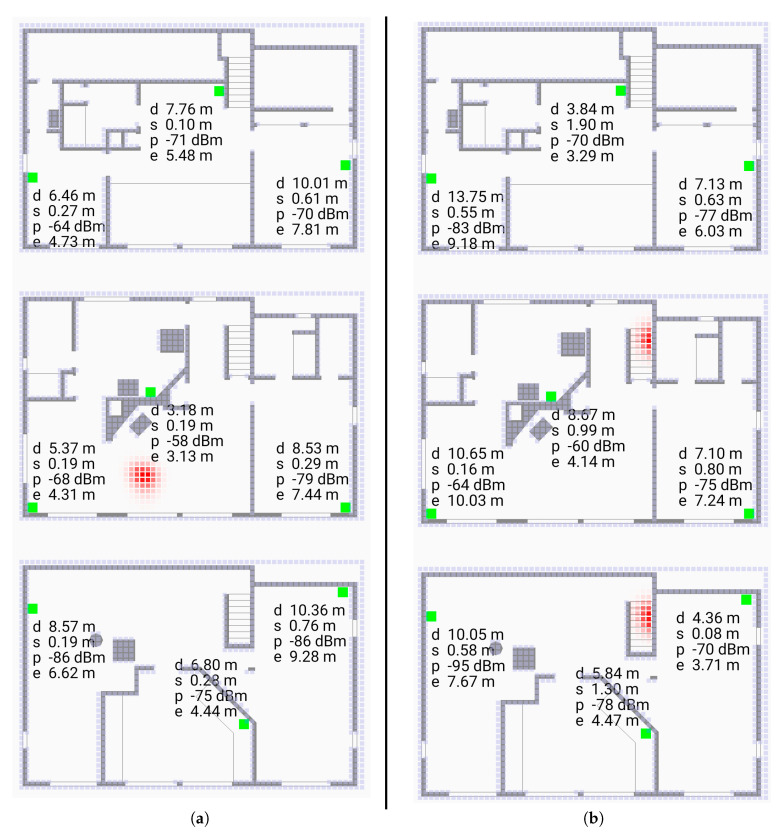
Screen shots of application using Bayesian grid update method as a “heat map” (from video [30]). The voxels are 0.2 m on a side. The green spots are the responders. (**a**) (left subfigure) initiator in middle of the middle floor (**b**) (right subfigure) initiator on stairs between floors. Note that the paths between initiator and responder in most cases pass through one or more walls or floors. The Bayesian grid update method uses the observation model developed here.

Next, we consider a more complex situation where position is recovered in three dimensions in a building with three floors. We show here that a parameterized “double exponential” model with a flat top fits real data well. Results of using this observation model in Bayesian cell updates are shown in Figure 11 (screen shots from the video [30]).

**Figure 12:**
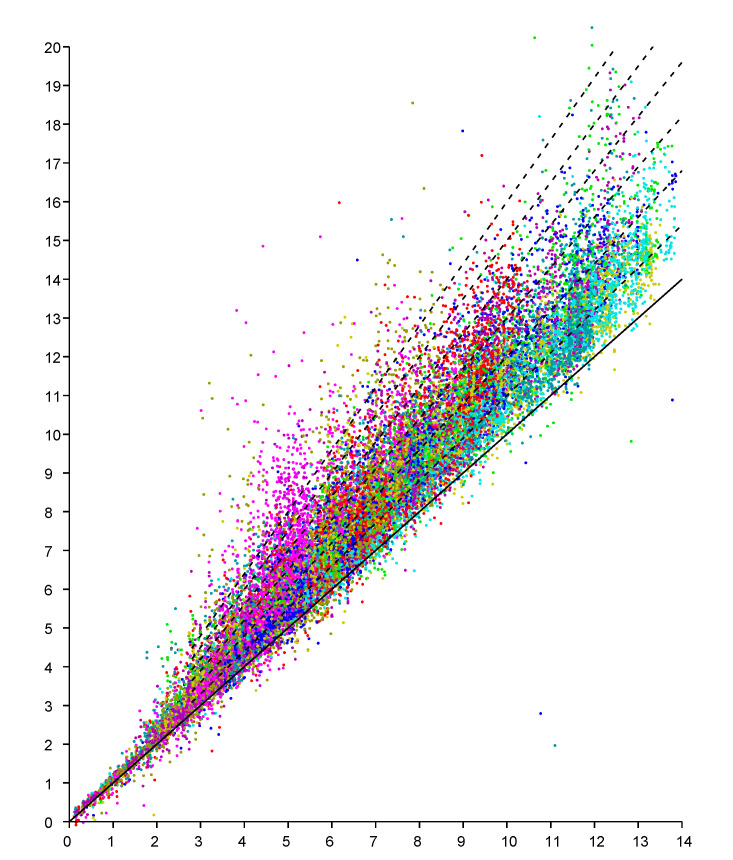
Scattergram of reported distance (vertical axis—meters) versus actual distance (horizontal axis—meters). Results from nine responders color coded. (Dashed lines have slope 1.0, 1.1, 1.2 *…* 1.6 ).

The scattergram in Figure 12 shows reported distance versus actual distance. Again, experiments suggest that the scattergram has structure that allows reduction of what appears to be a two-dimensional probability density distribution fitting problem to a one-dimensional one.

**Figure 13:**
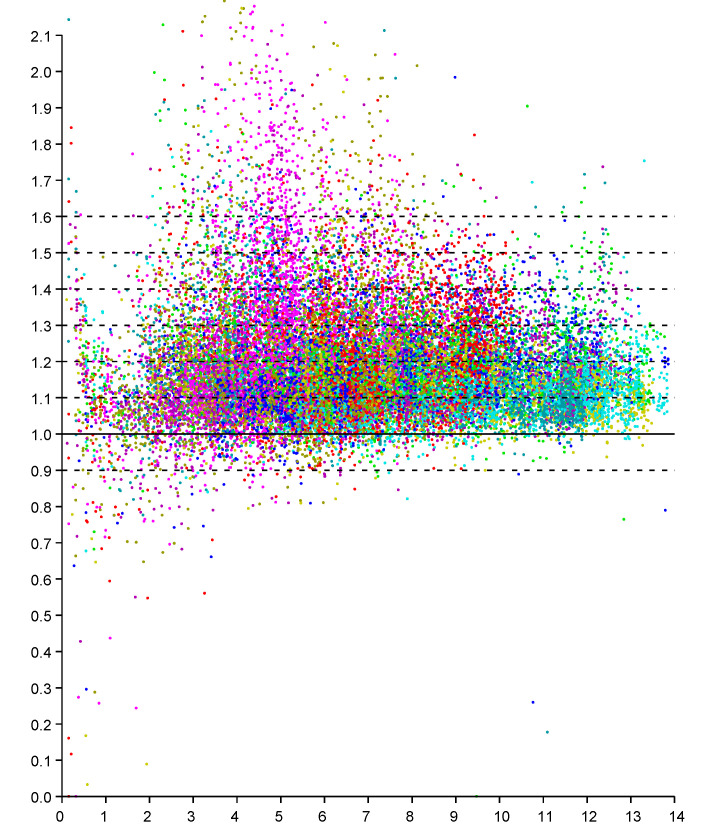
Scattergram of ratio of reported to actual distance (vertical axis) versus actual distance (horizontal axis—meters). Results from nine responders color coded. (Dashed lines are shown for ratios of 0.9, 1.0 1.1, 1.2 *…* 1.6).

This regularity becomes more obvious if we plot the *ratio* of reported distance to actual distance (versus actual distance). as in Figure 13. The vertical scatter in this diagram is more or less independent of actual distance (except perhaps for very small and very large distances). So we can collapse the two-dimensional scattergram into one were we plot the *ratio* of distances versus the actual distance.

**Figure 14:**
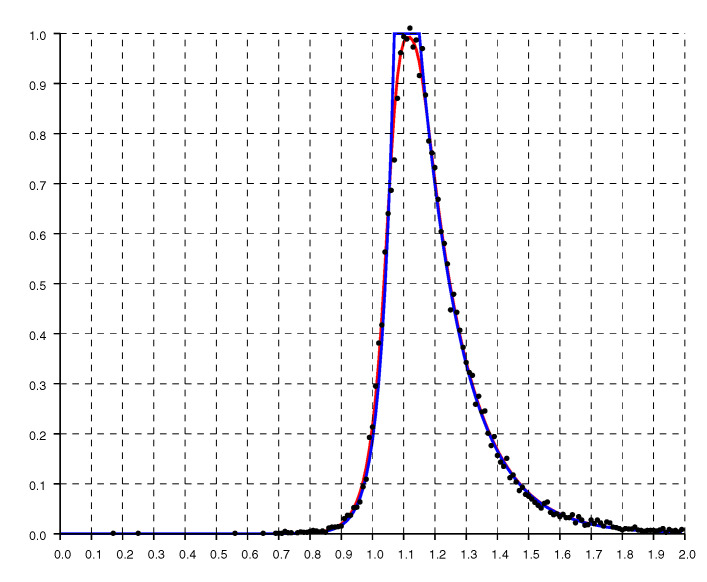
Histogram of the ratio of reported to actual distance based on about 20,000 measurements (scaled so that the peak equals one). The solid blue line is the “double exponential” with flat top parametric fit. The red curve is a smoothed version that takes into account measurement error with st. dev. of 0.25 m at 10 m.

Figure 14 shows a histogram of the ratio of reported to actual distance as a function of actual distance for about 20,000 measurements. These measurements are from three levels of a house of wooden stud and dry wall construction, with a brick chimney and metallic obstacles such as a large refrigerator, water heater, furnace, and a wood stove. The asymmetry is again apparent, with ratios greater than one occurring much more frequently than ratios smaller than one (we see that about 97% of the ratios are larger than one).

**Figure 15:**
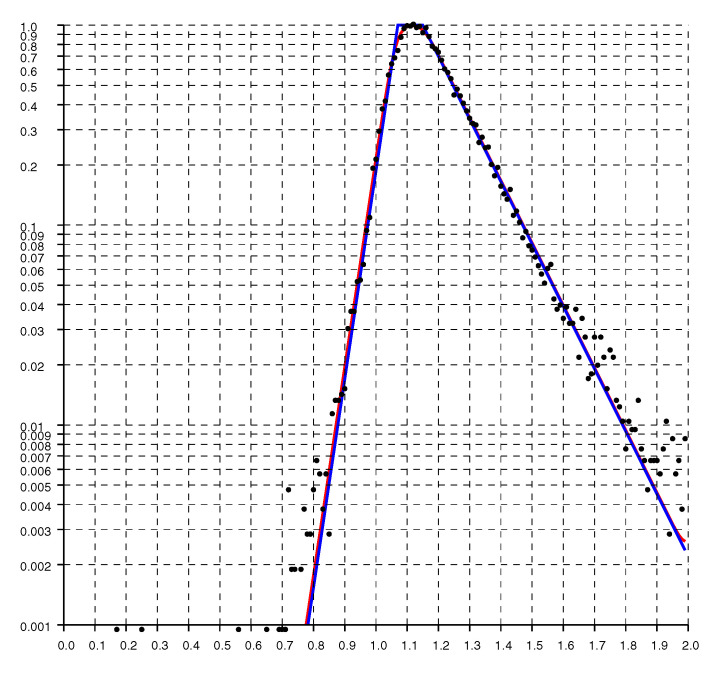
Histogram of the ratio of reported distances to actual distance on a logarithmic scale. The solid blue line is the “double exponential” with flat top parametric fit.

Replotting the histogram on a logarithmic scale, as in Figure 15, shows that the drop offs on either side of a flat top are exponential. As expected, the exponential decay is much more gradual on the left than on the right.

A model fit to the histograms from these experimental results is the “double exponential” with flat top

(4) $$h\left(r\right)=\frac{1}{H}\left\{\begin{array}{cc} e^{-\left(r_{l}-r\right)/s_{l}} & \mathrm{for}\;r<r_{l}\\ 1 & \mathrm{for}\;r_{l}\leq r\leq r_{r}\\ e^{-\left(r_{r}-1\right)/s_{r}} & \mathrm{for}\;r_{r}<r \end{array}\right.$$

where r=o/d is the ratio of, *o*, the reported (observed) distance, to, *d*, the actual distance. The distribution is constant in the region between the two decaying exponentials (i.e., for $r_{l}\leq r\leq r_{r}$). The “flat top” provides for a better fit in this more complex situation. It arises here because in this house the line of sight is rarely unobstructed and distance measurements go through varying amounts of material with high relative permittivity. For a start, in any given position, about 2/3 of the measurements are w.r.t. responders on other floors and thus go through one or two layers of flooring material.

**Figure 16:**
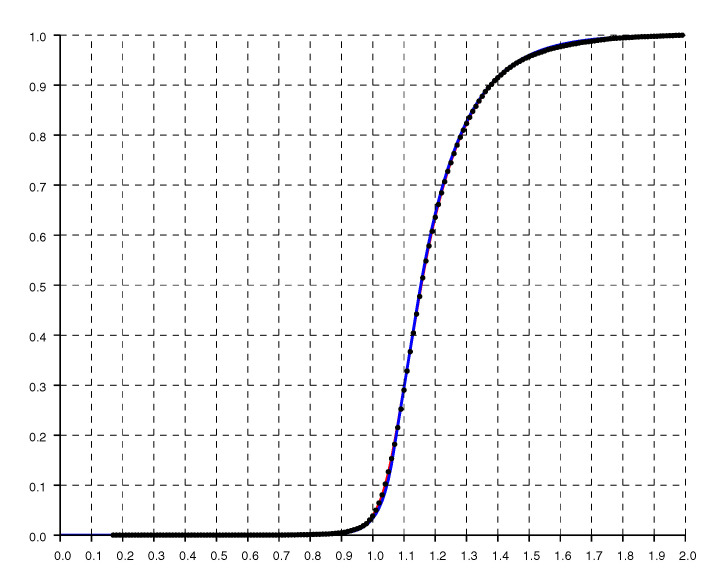
Normalized cumulative histogram of the ratio of reported to actual distance. The solid blue line is the “double exponential” with flat top parametric fit.

The constant $H=s_{l}+\left(r_{r}-r_{l}\right)+s_{r}$ normalizes the distribution so its integral (w.r.t. *r*) equals one. For the solid blue curves plotted in Figure 14, Figure 15 and Figure 16, $s_{l}=0.045$, and $s_{r}=0.136$, while $r_{l}=1.07$ and $r_{r}=1.17$.

Again, the conditional probability of reported distance, *o*, when the actual distance is *d*, is

(5) $$P\left(o|d\right)=\frac{1}{d}h\left(\frac{o}{d}\right)$$

Another check on the quality of the parametric fit is given by the cumulative distribution shown in Figure 16, where the dots are from experimental data while the solid blue curve is from the double exponential fit with flat top. About 97% of the ratios of measured to actual distance are larger than one.

The best-fit parameters, $s_{l}$, $s_{r}$, $r_{l}$, and $r_{r}$, estimated from the scattergram, depend somewhat on the structure of the building and the building materials. Fortunately, experiments indicate that they need not be known with great accuracy to get reasonable results with the Bayesian grid update method. For example. reasonable position determination is possible using the parametric model developed in Section 3 under different circumstances.

Note again, that, in practice, a weighted sum of this distribution and a uniform distribution should be used to account for the appearance of random outliers in the measurements.

## 7. Some Extensions and Some Limitations

The observation model is based on data collected in many positions for initiators and responders. As a result, it is a kind of average and does not apply exactly to any one particular combination of initiator and responder positions. We could compensate exactly for biased distance measurements if we had a complete model of a building, including wall and floor thicknesses as well as their electrical properties at radio frequencies. This is obviously not realistic. A well-tuned observation model is the best we can do *without* such detailed modelling.

It is not only building materials that have remarkably high relative permittivities. The human body does as well. Some tissues have relative permittivities as high as 40 to 60, which, not surprisingly, comes close to that of water (ε=85 at 5 GHz and room temperature) [31]. The relative permittivity of lungs is lower, perhaps 20 to 40 depending on how much air they contain [31]. The distance added by an obstacle of thickness *d* and relative permittivity ε is $d(\sqrt{\varepsilon }-1)$.

A person holding a phone at chest level adds about 1 to 1.5 m to the reported distances for responders that are behind them. This can easily be seen as one turns to face different directions when using an application that continuously reports FTM RTT measurements. Naturally this limits the position accuracy that can be attained. The impact of this effect is reduced if more than the minimum required number of APs is used.

## 8. Conclusions

Bayesian cell update has proven to be a promising method for estimating indoor position using FTM RTT distance measurements [21]. It requires an observation model that gives the conditional probability of observing a reported distance given an actual distance.

(1) The parameters of an observation model, to be used with the Bayesian cell update, may be fitted using scattergrams of reported distances versus actual distance. We have shown that scattergrams of real data indicate that the observation model fitting problem can be reduced from two dimensions to one.

(2) We have further observed that, perhaps surprisingly, a “double exponential” observation model fits real data well—particularly if we include a flat top with parameterized position.

(3) Generating test data involves knowing not only the positions of the responders (APs) but also that of the initiator. The effort required for manual determination of initiator positions can limit the scale of test data collection. We have shown here that “boot strapping” using results of a Bayesian grid update method as a proxy for the actual position works well.

(4) Indoors, reported distance measurements are biased to be mostly larger than actual distances. This bias can be detected. Unfortunately, a simple heuristic for directly compensating for the bias does not appear to lead to much better results (see Appendix A). As a result, it appears that the best approach is to use the Bayesian cell update method without this heuristic compensation.

(5) Overall, indoor positioning using FTM RTT has significant advantages over competing methods, including higher accuracy and the ability to work with lower spatial density of devices (see Appendix C).

## Figures and Tables

**Figure A1:**
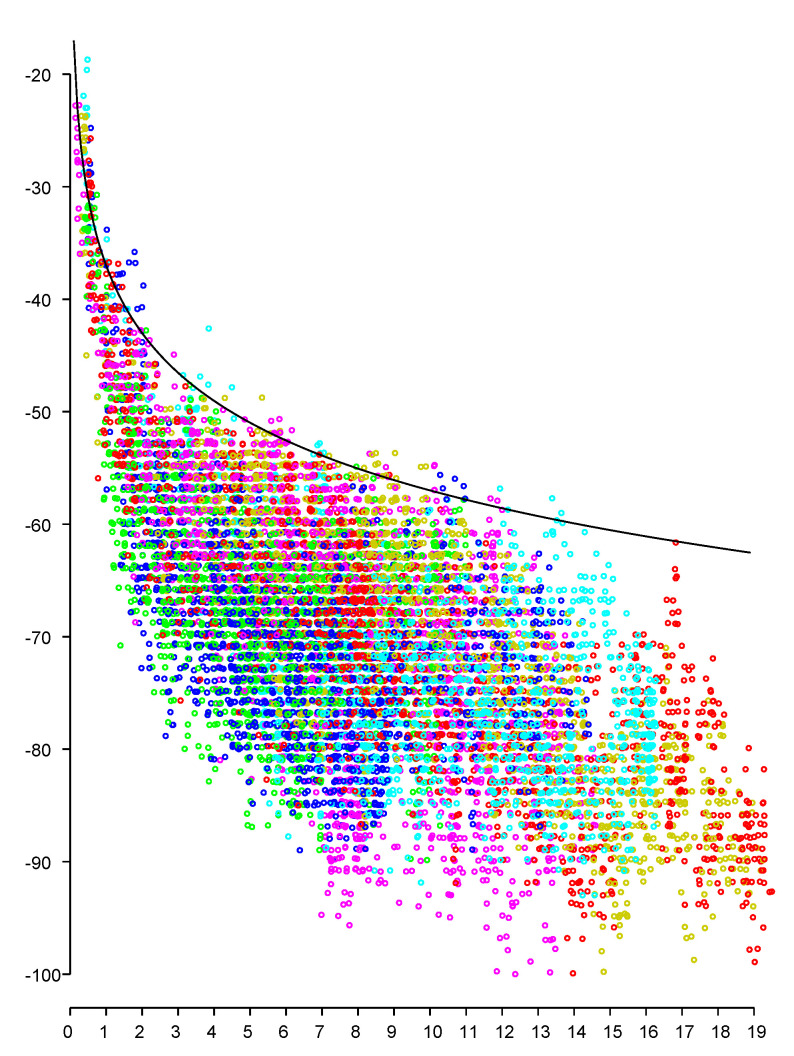
Scattergram of signal strength (vertical axis—dBm) versus actual distance (horizontal axis—meters) in typical house based on about 10,000 measurements. Results from six responders color coded. Solid curve is inverse square law prediction.

## Appendix A. Attempt at Partial Compensation for Bias in Distance Measurements

As we have seen, indoors, distance measurements from FTM RTT are biased due to delay in building materials of high relative permittivity and due to multi-path propagation when the line of sight is blocked [21]. These biases increase with distance but vary with circumstances. The magnitude of the effect depends on what obstacles the line connecting the initiator to the responder passes through. It would be useful to be able to detect the bias and attempt to partially compensate. We describe one heuristic for attempting to do this.

Suppose that *N* responders are at positions $\left\{x_{1},x_{2},\ldots x_{N}\right\}$ and the initiator at x. The actual distances are $\left\{d_{1},d_{2},\ldots d_{N}\right\}$ where $d_{i}=∥x-x_{i}∥$. We can compute these and compare them with the reported distances $\left\{r_{1},r_{2},\ldots r_{N}\right\}$ say. Typically, some, or all of the reported distances will be larger than the actual distances. This bias will tend to “push” a multilateration solution in directions away from the responders.

The effect is not so dramatic when the initiator is inside the convex hull of the responders, because the “forces” resulting from the biases will tend to be in different directions and partially cancel. For positions outside the convex hull, on the other hand, the estimated position will be displaced outwards, away from the centroid of the responder positions, with potentially larger errors (see also error ellipses in Figure 14 in [21]). Can we detect the bias and correct for it?

First, we can detect the bias by comparing the sum of squares of reported distances $\sum_{i=1}^{N}r_{i}^{2}$ with the sum of squares of actual distances $\sum_{i=1}^{N}d_{i}^{2}$ (As a proxy for the actual distances we can again use distances calculated based on the estimated position of the initiator, as described above). If there was no bias or measurement error, the two quantities would be equal. In the presence of the bias, however, the reported distances will generally be larger than the actual distances. We can calculate a compensating scale factor by relating the two:

(A1) $$s=\sqrt{\sum_{i=1}^{N}d_{i}^{2}/\sum_{i=1}^{N}r_{i}^{2}}$$

The expected value of this quantity would be 1 in the absence of bias (as e.g., outdoors). We can compensate for the bias by multiplying reported distances by *s* before using them in multilateration. The scaling will affect large distances more than smaller distances, which is as it should be, since measurements of large distances have larger biases.

One way to apply this heuristic is to first calculate the initiator position *without* compensation, then compute the scale factor *s* as above, and finally, recompute the initiator position using “compensated” distances. If the biases change slowly, a computationally more efficient approach would be to compute the scale factor as above, but use that scale factor with the *next* set of measurements.

While this method improves estimation of the position of the initiator in many cases, it also introduces new errors. This is because there is feedback between the estimated scale factor and the estimated position of the initiator, particularly when most responders are on one side of the initiator (exactly the situation were it would be useful to compensate for the bias).

There are some modifications of this approach that may be worth considering. One is to “put all the blame” on the largest measurement, since it is likely to have the largest bias. But in practice all measurements are affected and the larger ones, which are more biased, do have correspondingly larger adjustments with this approach anyway.

A related idea (suggested by one of the reviewers) is to actually *omit* one or more of the longest distance measurements since they are less reliable. This can help if the density of responders is high enough that they are not all needed to solve for the position (3 responders within range in 2-D, 4 responders in 3-D). Note that in the examples shown here (Figure 1 and Figure 11), the density of responders is higher than normally needed for indoor positioning because more responders were used for the collection of data for the histograms used in the fitting of probability densities.

In practice, there often wouldn’t be enough responders within range to make it possible to discard some measurements. Note, by the way, that the Bayesian update method “discounts” contributions from far away responders since the conditional probabilities for those are more spread out and smaller (see the right sides of Figure 7 and Figure 8).

Overall, unfortunately, this attempt at counteracting the bias, as it stands, has not proven to be very effective.

### Implications for Placement of Responders

It is interesting to consider how the quantity $\sum_{i=1}^{N}d_{i}^{2}$ varies with initiator position x. It is easy to show (by the parallel axis theorem) that it equals

(A2) $$N{\left(x-\bar{x}\right)}^{2}+I\left(\bar{x}\right)$$

where $\bar{x}$ is the centroid of the responder positions and

(A3) $$I\left(\bar{x}\right)=\sum_{i=1}^{N}{\left(x_{i}-\bar{x}\right)}^{2}$$

is the “inertia” of the responder positions about the centroid.

For the indoor position system to have similar accuracy in different part of its range, this sum should not vary a lot with initiator position x. So we want the ratio of ${\left(x-\bar{x}\right)}^{2}$ to $I(\bar{x})$ to be small. This means, first of all, that the centroid of the responders should be near the center of the area where the initiator may be found. Secondly the “inertia” should be relatively large. This means the responders should be spread out as far as feasible, not clustered near the centroid.

## Appendix B. Signal Strength Provides Only an Upper Limit

It is natural to wonder whether distance between the phone and the access point could be more easily estimated using the inverse square law applied to signal strength (RSS). Or, that signal strength could at least be a useful adjunct to FTM RTT distance measurements. A scattergram of signal strength versus distance in Figure A1 shows that this is not the case (see also [32]). Note that the signal strength for any particular distance can vary over a wide range (see also [33,34,35]). Similarly, the range of distances corresponding to a particular signal strength reading is large. For example, here, an RSS value of −60 dBm could correspond to distances anywhere between 1.5 m and 12 m.

For fixed transmitter power, signal strength does provide an *upper bound* on distance, as shown by the solid curve for

(A4) $$S=-37-20\log_{10}\left(d_{m}\right)$$

or

(A5) $$d_{m}=0.014\times 10^{-S/20}$$

This means that possible positions of the phone are restricted to a circle (sphere in 3-D case), rather than a circular annulus (spherical shell). Intersecting circles (or spheres) corresponding to different access points will in most cases lead to an area (volume) of considerable size and thus not pinpoint the positions of the phone well.

Further, the slope of the solid curve drops off with distance—the absolute accuracy of determining the upper bound $d_{m}$ drops inversely with distance (as can be seen by differentiating Equation (A4) with respect to distance).

Finally, the constant offset in Equation (A4) is for a particular transmitter power level, and APs change transmitter power depending on, for example, the level of interference.

## Appendix C. Efficiency of FTM RTT Compared to Beacons

How “efficient” is indoor positioning using FTM RTT with WiFi APs? That is, how many APs are needed to cover a certain area (2-D case) or a given volume (3-D case)? How does this compare to alternate indoor positioning schemes?

One alternate indoor positioning scheme depends on “beacons” (Bluetooth of WiFi) which can be detected at close range. A scattergram of signal strength versus distance such as Figure A1 shows that distance estimates cannot be made reliably from signal strength (see Appendix B). However, it *is* possible to tell when one is really close (say 1 m to 2 m) to one of the beacons. So one approach is to spread a large number of beacons, select the one with the strongest signal and use its position as the estimated position of the sensor. The accuracy of the result is just a function of how densely the beacons are packed.

In the 2-D case, we may first consider a simple square cell layout with beacons at the intersections of lines on a square grid. If the grid spacing is *g*, then to cover an area *A* one needs approximately $N=A/g^{2}$ beacons. When picking the position of the “loudest” source as the estimated position of the sensor, the largest error occurs when the sensor is right in the middle of one of the squares, and thus a distance $d=g/\sqrt{2}$ from the four corners. Thus, the required beacon density is

(A6) $$\frac{N}{A}=\frac{1}{2d^{2}}$$

For d=1 m, this comes to 0.5 beacons per square meter.

A layout based on square tessellation of the plane is, however, not the most efficient in this regard. Consider instead a layout based on a hexagonal tessellation, where there are again rows of beacons, now on a triangular grid, with odd numbered rows shifted by one half the beacon spacing to the right. In addition, the distance between rows is $\sqrt{3}/2$ times the spacing between beacons within a row. In this case, if the spacing between beacons is again *g*, then the number of beacons needed is $N=2A/(\sqrt{3}g^{2})$, while the largest error in position is $g/\sqrt{3}$. Thus

(A7) $$\frac{N}{A}=\frac{2}{3\sqrt{3}d^{2}}$$

which is about 30% more efficient than the simple square grid layout. For a desired accuracy of d=1 m, the density is 0.384 beacons per square meter for this hexagonal arrangement.

As we saw, the method based on FTM RTT is good out to about 10 m to 20 m, with about 1 m accuracy (if a good observation model is used) and needs three to six responders to cover an area of 100 to 200 m${}^{2}$ (Figure 1). That is, a density of about 0.030 responders per square meter.

Thus, in this regard, FTM RTT indoor positioning is considerably more efficient than a method based on beacons. On the other hand, RF beacons can be simpler and cheaper than WiFi APs. Still, installing, powering, locating, and maintaining a large number of beacons involves considerable effort.

The above analysis is for the “2-D” case of a single level in a building. Multiple levels may be treated as separate 2-D problems or as full “3-D” problems.

If we place beacons on the corners of a cubic grid of spacing *g*, we need about $N=V/g^{3}$ beacons for a volume *V*. The largest error will be when the sensor is in the middle of one of the cubes and thus $d=\sqrt{3}g/2$ from the eight corners. So

(A8) $$\frac{N}{V}=\frac{3\sqrt{3}}{8d^{3}}$$

For d=1 m, this comes to 0.65 beacons per cubic meter.

We can do better with different layouts. Starting with the grid based on hexagonal cells described above for the 2-D case, add a layer above it shifted by $(1/2,1/\left(2\sqrt{3}\right),\sqrt{2/3})g$ and so on (this corresponds to a hexagonal close-packing arrangement). It can be shown that in this case $N=\sqrt{2}V/g^{3}$.

Next, the point $(1/2,1/\left(2\sqrt{3},1/2\sqrt{2/3}\right)g$ is a distance $d=\sqrt{3/8}g$ from (twelve) neighboring beacons, which is as far away as it can be with this layout. So

(A9) $$\frac{N}{V}=\frac{3\sqrt{3}}{16d^{3}}$$

which is twice as good as with the simple cubic arrangement. For d=1 m, this comes to 0.325 beacons per cubic meter.

As we saw, the method based on FTM RTT needs four to nine responders to cover a volume of 500 to 700 m${}^{3}$ (Figure 11). This is a density of about 0.008 responders per cubic meter.

Thus once again, FTM RTT indoor positioning is considerably more efficient than a method based on beacons. One may argue that resolution in the vertical directions is not as important, and so a beacon layout with coarser spacing in the vertical direction could be used without much loss in accuracy. At the same time, figuring out which floor a person in need of help is in an emergency is important and an error larger than 1 meter could make that determination less reliable.

## Appendix D. Convolution of Double Exponential with the Probability Distribution of the Measurement Noise

In order to account for additive measurement errors in the observation model, we should convolve the double exponential with the distribution of the measurement errors.

Consider a Gaussian distribution for the measurement errors. The convolution of the one-sided decaying exponential $f_{s}\left(x\right)$

(A10) $$f_{s}\left(x\right)=\left\{\begin{array}{cc} e^{-x/s} & x\geq 0\\ 0 & \mathrm{otherwise} \end{array}\right.$$

and the Gaussian

(A11) $$g_{\sigma }\left(x\right)=\frac{1}{\sqrt{2\pi }\sigma }e^{-\frac{1}{2}{\left(x/\sigma \right)}^{2}}$$

is

(A12) $$h_{\sigma ,s}\left(x\right)=\frac{1}{2}e^{\frac{1}{2}{\left(\sigma /s\right)}^{2}-x/s}\;\mathrm{erfc}\left(\frac{1}{\sqrt{2}}\left(\frac{\sigma }{s}-\frac{x}{\sigma }\right)\right)$$

where erfc(…) is the complementary error function, which can be defined in terms of the error function erf(…) by

(A13) erfc(x)=1−erf(x)

Numerical implementation of Equation (A12) is tricky, since, when *x* becomes large and negative, the first term in the product becomes very large, while the second becomes very small.

Fortunately, another formula is obtained if we re-express the above using the *scaled* complementary error function

(A14) $$\mathrm{erfcx}\left(x\right)=e^{x^{2}}\mathrm{erfc}\left(x\right)$$

We obtain

(A15) $$h_{\sigma ,s}\left(x\right)=\frac{1}{2}e^{-\frac{1}{2}{\left(x/\sigma \right)}^{2}}\;\mathrm{erfcx}\left(\frac{1}{\sqrt{2}}\left(\frac{\sigma }{s}-\frac{x}{\sigma }\right)\right)$$

Numerical implementation of Equation (A15) is also tricky, since, when *x* becomes large and positive, the second term in the product becomes very large, while the first term becomes very small.

For accurate results we need both formulae: use Equation (A12) when $x\geq \sigma^{2}/s$; and use Equation (A15) when $x<\sigma^{2}/s$. Note that at the transition between the two cases, i.e., for $x=\sigma^{2}/s$, both formulae yield

(A16) $$\left(1/2\right)e^{-\frac{1}{2}{\left(\sigma /s\right)}^{2}}$$

since erfc(0)=1 and erfcx(0)=1. To implement this in practice, we need robust numerical approximation of both erfc(…) and erfcx(…). See e.g., [36].

Finally, we add the contributions from the two exponentials

(A17) $$h_{\sigma ,s_{l}}\left(r_{l}-x\right)+h_{\sigma ,s_{r}}\left(x-r_{r}\right)$$

If there is a “flat top” (i.e., if $r_{r}\neq r_{l}$), we have to add the contribution from that as well. The convolution of the Gaussian $g_{\sigma }\left(x\right)$ with the “flat top “ pulse $f_{s_{l},s_{r}}\left(x\right)$, comes to

(A18) $$\frac{1}{2}\left(\mathrm{erf}\left(\frac{r_{r}-x}{\sqrt{2}\sigma }\right)-\mathrm{erf}\left(\frac{r_{l}-x}{\sqrt{2}\sigma }\right)\right)$$

While the distribution of measurement errors is not Gaussian, the result of the convolution will not be affected much by the exact shape of the assumed distribution. The convolution has little effect on the distribution for larger actual distances, but it does smooth out sharp peaks in the conditional probabilities that would occur otherwise for very small actual distances.
